# Response: Commentary: Outlining a novel psychometric model of mental flexibility and affect dynamics

**DOI:** 10.3389/fpsyg.2025.1631344

**Published:** 2025-10-01

**Authors:** Francesca Borghesi, Alice Chirico, Pietro Cipresso

**Affiliations:** ^1^Department of Psychology, University of Turin, Turin, Italy; ^2^Department of Psychology, Research Center in Communication Psychology, Universitá Cattolica del Sacro Cuore, Milan, Italy

**Keywords:** Mental Flexibility, Cognitive Flexibility, affect, stability, variability, psychometrics

## Introduction

We thank Ionescu and Gros for their thought-provoking commentary, which continues an important discussion on the conceptual foundations of Flexibility. The term *Flexibility* has long suffered from fragmentation across domains—cognitive, behavioral, affective, and psychological—leading to inconsistent operationalizations and reduced theoretical clarity. Cognitive Flexibility, in particular, is often treated either as the core construct or as entirely distinct from broader forms such as behavioral regulation or experiential openness in therapeutic models like ACT. In our model, we propose *Mental Flexibility* as a unifying, overarching construct that integrates these diverse expressions. Defined as an emergent capacity for adaptive variability, Mental Flexibility accounts for both changes in behavior and the maintenance of self-coherence across shifting contexts. Due to Flexibility's strong connection to the concept of variability, which involves shifting or changing, our framework also explores its interplay with affect dynamics, highlighting how shifts in cognition and behavior correspond to emotional fluctuations. The critiques addressed here pertain to conceptual clarity, measurement issues, and our theoretical positioning within the variability-stability-flexibility continuum.

### Response to critique on mechanism, ability, and the conceptual framing of mental flexibility

Ionescu and Gros (2025) argue that conceptualizing Mental Flexibility as a property is incompatible with its association with mechanisms, skills, or abilities. They maintain that these are distinct categories and suggest that our use of Mental Flexibility lacks originality. However, we contend that these levels—property, mechanism, skill—are not mutually exclusive but represent interconnected layers of abstraction, like in intelligence or consciousness (Gamez, 2020; Kanai and Fujisawa, 2024).

As an emergent property, Mental Flexibility arises from the coordinated activity of sub-processes and not reducible to their components (de Schotten and Forkel, 2022), yet they require mechanistic elucidation (Miller et al., 2024). Mechanisms such as shifting, inhibition, updating, and control (Illari and Williamson, 2012; Machamer et al., 2000) provide the operational basis for Flexibility, just as encoding and retrieval support memory. These mechanisms are embedded within broader systems like cognitive control and episodic memory (Egner, 2023). Skills and abilities describe how such mechanisms manifest behaviorally, evolving from effortful to automatized processes (Ackerman, 1992; Hambrick et al., 2018; Sheffler et al., 2022). Hence, Flexibility manifests as both ability and trait (Zhang et al., 2020). Performance tasks assess momentary flexibility, based on time response of switching cost (Demanet et al., 2011; Grol and De Raedt, 2018; Howell and Hamilton, 2021), while self-reports capture dispositional tendencies (Bond et al., 2011; Dennis and Vander Wal, 2010; Gabrys et al., 2018; Martin and Rubin, 1995; Rogge and Lin, 2024). Low correlations between methods are common across constructs—such as Intelligence, Empathy, Creativity—where different behavioral measures tap into the same underlying trait but remain weakly correlated (Costa and Faria, 2020; Decety, 2011; Howlett et al., 2021, 2022; Kandler et al., 2016). Finally, in our conceptualization, Mental Flexibility is not a synonym for Cognitive Flexibility as in Anziano et al. (2023); it is a superordinate construct uniting cognitive, affective, behavioral, and psychological domains, re-anchored within psychological science. Throughout our conceptual work, we used the terms *Flexibility* and *Mental Flexibility* interchangeably, as both refer to an emergent property or meta-function involved in cognitive and affective shifting or switching processes. The addition of the adjective *Mental* was a deliberate choice to prevent confusion with uses of Flexibility in other scientific domains—such as material science, chemistry, physics (Bruns et al., 2020), logistic organization of workplace (Manders et al., 2017) and motor functional ability (Stathokostas et al., 2012). Rather than being redundant, this specification serves a clarifying and boundary-setting function within psychological science. It aims to identify overarching features that may unify the various domain-specific definitions of Flexibility (e.g., cognitive, affective, behavioral, and psychological flexibility). These definitions remain distinct and autonomous, but the goal is to facilitate integration by highlighting their shared, higher-order characteristics-connections that, until now, have rarely been conceptually aligned.

### Methodological considerations and the use of Markov chains

Ionescu and Gros (2025) criticize the omission of several classical tasks—such as the Dimensional Change Card Sort (DCCS) (Zelazo, 2006), Navon task (Kimchi, 1992; Navon, 2003), Plus-Minus (Miyake et al., 2000), and Brixton test (Spitoni et al., 2018)—from our framework, and our reliance on self-report measures. Our article, however, did not aim to review all flexibility tools, but to present a novel model integrating cognitive, affective, and behavioral dimensions within affect dynamics. We included representative direct and indirect measures to illustrate a key point: tools labeled as Flexibility measures often show weak intercorrelations (Fang and Ding, 2022), underscoring the need for a more integrated model. The excluded tasks are valid but context-specific: DCCS is optimized for preschoolers; Navon targets low-level perceptual shifts, not executive flexibility. Plus–Minus and Brixton assess rule-shifting mechanisms already covered by other tools in our model. Even recent reviews (Hohl and Dolcos, 2024) mention only the DCCS from Ionescu's list.

Regarding Discrete Time and Space Markov chains, we framed them as a heuristic for modeling affective transitions (Borghesi et al., 2025; Hamaker et al., 2015), not as a validated tool. Inspired by socioemotional flexibility models (Hollenstein, 2015), we propose that affective variability may reflect Mental Flexibility. Although our initial work utilizes self-report data, the model is extendable to performance-based tasks and warrants further empirical refinement.

### Dynamic models and the interplay of variability, stability, and flexibility

Ionescu and Gros (2025) critique our conceptualization of Mental Flexibility as an emergent property characterized by *adaptive variability*, favoring instead a developmental continuum from variability to stability and then flexibility (Ionescu, 2017). However, empirical evidence suggests this progression is neither linear nor universally adaptive. In Blakey et al. (2016), children move from mixed responding to perseveration—not directly to flexibility—highlighting that variability is not random, and perseveration does not represent functional stability. Similarly, Event-Related Potentials (ERPs) studies show that infants exhibit systematic responses to phonological prediction errors (Ylinen et al., 2017), supporting predictive coding models (Millidge et al., 2021), where behavior is guided by internal models that minimize prediction error (van de Cruys et al., 2014). This finding aligns with predictive coding theories, which propose that behavioral variability is structured and guided by internal models continuously updated to minimize prediction error. Supporting this, Gopnik et al. (2017) liken children's exploration to simulated annealing algorithms, reflecting strategic, inference-driven behavior. Defeyter and German (2003) similarly show that children's openness to noncanonical object use stems from Cognitive Flexibility, not random variability. These findings challenge Ionescu's developmental continuum by showing that early variability is structured, purposeful, and indicative of an already flexible and exploratory cognitive system.

Nonetheless, the proposal by Ionescu and Gros fits within a broader discussion regarding the relationship between stability and flexibility. Traditionally, many scholars have embraced a stability–flexibility trade-off model, wherein cognitive stability (i.e., task focus, resistance to distraction) and flexibility (i.e., task-switch readiness) are conceptualized as opposing ends of a single continuum. According to this model, enhancing stability necessarily reduces flexibility, and viceversa, due to a shared control parameter—often described as an “updating threshold” within working memory (Dreisbach et al., 2024; Goschke and Bolte, 2014; Hommel and Colzato, 2017).

Our conceptualization draws from this flourishing debate, shifting the focus toward the trade-off between stability and variability—an approach that aligns with statistical frameworks commonly used in dynamic analysis (Del Giudice and Crespi, 2018; Geddert and Egner, 2022). We conceptualize Mental Flexibility as a meta-property that dynamically balances variability and stability in response to internal goals and contextual demands. Flexibility, in this sense, is not mere reactivity but involves intentional modulation—choosing stability when consistency is required and shifting when adaptation is needed. This *adaptive variability* reflects a goal-directed form of switching, rather than random change, and is closely tied to personal meaning and self-regulation. The capacity for variability is typically assessed through switching costs in neuropsychological tasks, and through self-report measures that emphasize the exploration of alternatives, such as shifts in perspective and behavior. Conversely, the capacity for stability, often neglected in literature, is reflected in self-report instruments like the Cognitive Flexibility Inventory (CFI) (Dennis and Vander Wal, 2010), the Cognitive Control and Flexibility Questionnaire (Gabrys et al., 2018) (CCFQ – Control subscale), the Coping Flexibility Scale (Co-Flex—Reflection component) (Vriezekolk et al., 2012), and the Multidimensional Psychological Flexibility Inventory (MPSI—Acceptance and Values subscales) (Rolffs et al., 2018). Stability also emerges in performance-based tasks through the analysis of switching costs, which quantify the cognitive effort required to maintain vs. shift responses across trials.

Ionescu and Gros (2025) question our use of “adaptability”, suggesting it conflates behavioral adjustment with a loss of authenticity. However, our references to Chen and Tang (2022) were intended to highlight that adaptation may sometimes reflect *reactive* rather than *agentic* strategies—such as “avoidance crafting,” which is driven by threat appraisals and may lead to disengagement. Similarly, our use of O'Toole et al. (2020) aimed to emphasize the role of emotional complexity in promoting authentic, context-sensitive adaptation: “*The ability to experience and distinguish multiple emotions can help inform more nuanced and flexible responses.”*

## Discussion

Our model thus distinguishes between superficial adaptation and deep, emotionally integrated Flexibility. It treats Flexibility not as a midpoint between extremes but as a meta-function coordinating transitions between stability and variability across domains-cognitive, affective, behavioral, and psychological.

Mental Flexibility offers a clear, integrative visualization of Mental Flexibility as the dynamic regulation between stability and variability over time. The U-shaped curve could represent Flexibility as optimal when both behavioral change and duration remain within a balanced range. At the two extremes, Flexibility collapses: inertia arises when the system remains overly stable for too long (minimal change), while instability reflects prolonged, uncontrolled variability (excessive change).

The central zone—labeled the *normal spectrum of Flexibility—*illustrates where adaptive functioning occurs. Here, the individual can oscillate between stable and variable behaviors depending on internal goals and contextual demands. The central vertical marker further highlights that Flexibility is not about avoiding change or pursuing it blindly, but about selectively and meaningfully regulating one's position along this continuum.

Our reconceptualization supports a multidimensional view of Flexibility and opens practical avenues for profiling patterns—rigid, disorganized, or adaptively flexible—to guide interventions such as Acceptance and Commitment Therapy (ACT) or emotion regulation training. It invites future research to develop assessments that bridge momentary performance and long-term dispositional flexibility across domains.
